# Electrocatalysts for Oxygen/Hydrogen-Involved Reactions

**DOI:** 10.3390/molecules27092628

**Published:** 2022-04-19

**Authors:** Jingqi Guan, Yin Wang

**Affiliations:** 1 Institute of Physical Chemistry, College of Chemistry, Jilin University, 2519 Jiefang Road, Changchun 130021, China; 2 Inner Mongolia Key Laboratory of Carbon Nanomaterials, Nano Innovation Institute (NII), College of Chemistry and Materials Science, Inner Mongolia Minzu University, Tongliao 028000, China

Oxygen/hydrogen-involved reactions are key reactions in many energy-related technologies, such as electrolytic water, electrocatalytic carbon dioxide reduction, electrochemical ammonia synthesis, rechargeable metal–air batteries, and renewable fuel cells. The development of highly active and stable hydrogen evolution reaction (HER), oxygen evolution reaction (OER), and oxygen reduction reaction (ORR) electrocatalysts is key to the large-scale implementation of these technologies. In addition, novel, high-performance HER, OER, and ORR catalysts could be predicted by theoretical investigations. This Special Issue aims to provide a broad survey of the most recent advances in oxygen/hydrogen-involved reactions and the applications in proton exchange membrane fuel cells (PEMFCs) and lithium-ion batteries (LIBs).

In this Special Issue, nine original research articles containing some recent experimental and theoretical advances in HER, OER, and ORR are reported. Five articles deal with the design and application of electrocatalysts for oxygen and hydrogen evolution reactions. Zhou et al. reported a 2D SnSe film in situ grown on a mica substrate using the molecular beam epitaxy method. The defective Sn structure was modulated by controlling experiment conditions and a p-type semiconductor of SnSe was formed. First-principles calculations revealed that Sn vacancies can serve as reactive centers and promote electron migration for HER [1]. Song et al. synthesized PtNi alloy loaded on zeolite imidazolate framework (ZIF), derived from porous nitrogen-doped carbon, and found that the optimized PtNi/MNC-1-6 catalyst exhibited a comparable electrocatalytic HER activity with that of commercial 20 wt% Pt/C and showed a quite small Tafel slope of 21.5 mV dec^−1^ [2]. Sondermann et al. reported a series of NiM-BTC (M = Co, Fe, BTC = 1, 3, 5-benzanetricarboxylate) electrocatalysts for alkaline OER. They found that Ni_10_Co-BTC and Ni_10_Co-BTC/KB composites underwent structural changes at high potentials, and the generated Ni(OH)_2_ contributed to the high electrocatalytic performance and stability of the OER [3]. Liu et al. developed a novel Sm_0.5_Sr_0.5_Co_0.8_Ni_0.2_O_3-__δ_ (SSCN82) nanofiber structure compound as a bifunctional electrocatalyst for OER and ORR, which afforded an onset potential of 1.39 V for OER and a Tafel slope of 111.8 mV dec^−1^ for ORR [4]. Woitasske et al. reported a facile microwave-induced heating method to prepare Pt nanoparticles on reduced graphite oxide (Pt-NP@rGO). HRTEM revealed that 2 nm~6 nm Pt particles were homogeneously distributed on the rGO without aggregation, which showed superior catalytic activity for the ORR [5]. Two articles deal with computational studies of HER, ORR, and OER electrocatalysts. Cherif et al. used density functional theory (DFT) to study the effects of fluorine modification of FeN_x_-doped and N-doped carbon catalysts on the ORR performance for PEM fuel cells. They studied 12 possible FeN_x_-doped and N-doped carbon models in the absence or presence of fluorine atoms. The results showed that metal-free catalytic N-sites were difficult to combine with F atoms, and in FeN_x_-doped carbon configuration, only one F atom coordinated with the FeN_x_ moiety helped to improve its ORR activity [6]. Yang et al. calculated the electrocatalytic thermodynamics of HER, OER, and ORR on three two-dimensional, Fe-based, metal–organic frameworks (2D Fe-MOFs). They investigated NH, O, and S ligands influencing the electronic structure and catalytic performance of 2D-Fe-MOFs and confirmed that Fe-O MOF displayed an optimal △G_H_ of 0.008 eV for HER, Fe-NH MOF exhibited the lowest η_ORR_ of 0.38 V for ORR, and three Fe-MOFs possessed poor OER performance (η_OER_ > 0.92 V) [7]. Two articles study the design of electrodes for use in fuel cells and batteries. Song et al. investigated the degradation of Pt electrocatalyst in PEMFCs under conditions of high energy efficiency. The corrosion of carbon support and the agglomeration of Pt nanoparticles under high operation potential (0.8 V) and high current density (1000 mA cm^−2^) led to the cell voltage declining more than 13 % after the operation for 64 h [8]. Wang et al. reported a nanosheet-assembled SnO_2_-integrated anode for LIBs on which a large reversible specific capacity of 637.2 mAh g^−1^ could be achieved. Such excellent capacity was a benefit of the rational design based on structural engineering to boost the synergistic effects of the integrated electrode [9].

In conclusion, oxygen/hydrogen-involved reactions are undoubtedly a hot and crucial topic in energy-related fields. Several research groups have contributed to the advancement of this topic by designing novel heterogeneous HER, OER, and ORR electrocatalysts, characterizing their structure, evaluating the catalytic performance, and studying the structure–activity relationship. Herein, important advancements in oxygen/hydrogen-involved reactions are revealed. We thank all of the authors for their valuable contributions to this Special Issue, all the peer reviewers for their valuable comments, criticisms, and suggestions, and the staff members of MDPI for the editorial support.
